# Possible antidepressant mechanisms of omega-3 polyunsaturated fatty acids acting on the central nervous system

**DOI:** 10.3389/fpsyt.2022.933704

**Published:** 2022-08-31

**Authors:** Lie Zhou, Jia-Yao Xiong, Yu-Qian Chai, Lu Huang, Zi-Yang Tang, Xin-Feng Zhang, Bo Liu, Jun-Tao Zhang

**Affiliations:** ^1^Yangtze University Health Science Center, Jingzhou, China; ^2^Mental Health Institute of Yangtze University, Jingzhou, China; ^3^Jingzhou Mental Health Center, Jingzhou, China

**Keywords:** omega-3 PUFAs, depression, neurotransmitter systems, neuroplasticity, synaptic plasticity, neuroinflammation, neurodegeneration, HPA axis

## Abstract

Omega-3 polyunsaturated fatty acids (PUFAs) can play important roles in maintaining mental health and resistance to stress, and omega-3 PUFAs supplementation can display beneficial effects on both the prevention and treatment of depressive disorders. Although the underlying mechanisms are still unclear, accumulated evidence indicates that omega-3 PUFAs can exhibit pleiotropic effects on the neural structure and function. Thus, they play fundamental roles in brain activities involved in the mood regulation. Since depressive symptoms have been assumed to be of central origin, this review aims to summarize the recently published studies to identify the potential neurobiological mechanisms underlying the anti-depressant effects of omega-3 PUFAs. These include that of (1) anti-neuroinflammatory; (2) hypothalamus-pituitary-adrenal (HPA) axis; (3) anti-oxidative stress; (4) anti-neurodegeneration; (5) neuroplasticity and synaptic plasticity; and (6) modulation of neurotransmitter systems. Despite many lines of evidence have hinted that these mechanisms may co-exist and work in concert to produce anti-depressive effects, the potentially multiple sites of action of omega-3 PUFAs need to be fully established. We also discussed the limitations of current studies and suggest future directions for preclinical and translational research in this field.

## Introduction

Depression is a mental disorder characterized by the sadness, loss of interest in activities, and decreased energy. It is often accompanied by the cognitive impairment and different physical symptoms. In severe cases, it may lead to suicidal tendencies (1). Depression is a high-incidence mental illness that has affected more than 264 million people of all ages worldwide by 2021 (2). Subclinical depression has a higher incidence in the general population. The rate of subclinical depression can reach up to 17% in the primary care and community setting (3, 4). Moreover, the rate for the high school students is 22.9%, while that for the college students have reached 36.56%. Most currently available antidepressants can target monoamine neurotransmitter function. However, current pharmacological treatments of depression suffer from major problems, such as a low rate of response, slow onset of therapeutic effects, loss of efficacy over time, and serious side effects. Therefore, development of novel strategies both for the prevention and treatment of depression has become increasingly important in today's medical field.

Omega-3 polyunsaturated fatty acids (PUFAs) are currently an attractive candidate for the prevention and treatment of depressive symptoms (5, 6). Being an essential nutrient, humans cannot synthesize omega-3 PUFAs *de novo*, therefore, these fatty acids must be obtained through diet or supplementation. Fatty acids are the most abundant organic compounds in the brain, making up 60% of the dry weight, among which 20% of these fatty acids are PUFAs. The two most abundant PUFAs in the brain are omega-3 docosahexaenoic acid (DHA, C22:6 ω-3) and omega-6 PUFAs arachidonic acid (AA, C20:4 ω-6) (7). Brain function is heavily dependent on adequate omega-3 PUFAs levels. Omega-3 PUFAs, mainly DHA and eicosapentaenoic acid (EPA) which have strong anti-inflammatory and inflammation-resolving effects, also antagonizing the pro-inflammatory effects of omega-6 PUFAs which are the precursors of pro-inflammatory mediators. The balance between omega-6 PUFAs and omega-3 PUFAs is essential for homeostasis and the proper functioning of the central nervous system (CNS) to promote mental health and prevent neurological diseases (7, 8). Since omega-3 PUFAs and omega-6 PUFAs compete for incorporation into cell membranes, a balanced intake of these different type of PUFAs is essential (8). In modern society, human diets are unbalanced between omega-3 PUFAs and omega-6 PUFAs that may restrict the supply of omega-3 PUFAs to the tissues leading to a mild or severe omega-3 PUFAs deficiency in both developed and developing countries worldwide (9–12).

As an integral component of cell membranes, omega-3 PUFAs can increase membrane fluidity and permeability. Omega-3 PUFAs are largely esterified to the phospholipid in the cell membrane. Once omega-3 PUFAs are released from the membrane following neurotransmitter receptor-mediated activation of specific phospholipase A2 (PLA2) enzymes, they can act as secondary messengers and regulate signal transduction, either directly or indirectly by their bioactive derivatives (13, 14). Omega-3 PUFAs and their derivatives regulate various processes within the CNS, such as neuroinflammation, neurotransmission, synaptic plasticity, neurogenesis, neurodegeneration, and thereby mood and behavior. Omega-3 PUFAs deficiency are associated with many neurological disorders, including Alzheimer's disease, major depression and anxiety disorder (7, 14). There is a substantial body of evidence that provides general support for the beneficial effects of omega-3 PUFAs supplementation on brain structure and function in healthy human subjects (15).

There is a considerable amount of literature regarding the mechanism of action of omega-3 PUFAs to improve physical health and brain functioning [see, e.g., (8, 14, 16–18)], and many of them have been further considered as possible mechanisms for omega-3 PUFAs to improve depressive symptoms. Omega-3 PUFAs supplementation in depressed subjects has many structural and functional benefits for the brain, including promoting neurogenesis and neural repairment, preventing neuroinflammation and neurodegeneration, improving mood, cognition and memory etc., thus, exerting a wide range of ameliorating effects on depression (19–21). They are nutritious and safe, and when used in combination with other antidepressants, they can accelerate and increase efficacy significantly (5).

However, the antidepressant mechanisms of omega-3 PUFAs acting on the CNS are still not fully understood. Nowadays, there is limited data on human brain with respect to the antidepressant effect of omega-3 PUFAs. This paper, for the first time, makes a systematic review about the research progress in this field over the last decade, starting with the cellular and molecular basis of omega-3 PUFAs. This will provide information reference for the future research and clinical practice.

## The cellular and molecular basis of omega-3 PUFAs

**Figure 1:**
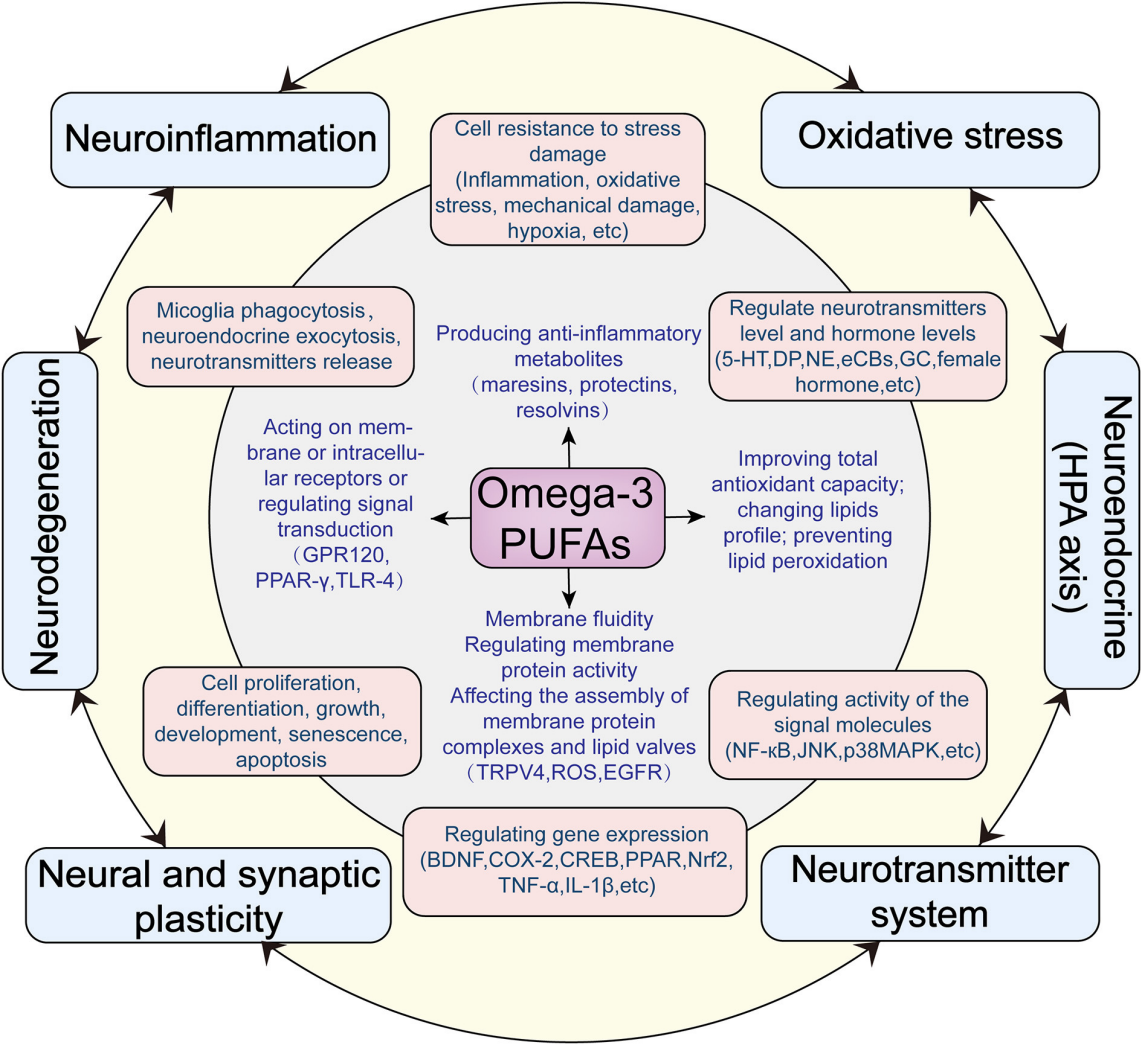
Hypothesized antidepressant mechanisms of omega-3 PUFAs acting on the central nervous system.

As shown in Figure 1, omega-3 PUFAs have a wide range of effects at the molecular and cellular levels, which may produce profound influences on mental health. We now summarize the main effects as follows:

### Increasing cell membrane fluidity and lipid bilayer elasticity, thereby influencing the structure of lipid microdomains, the interaction and function of proteins (including receptors, channel proteins, enzymes) in the membrane

Through optimizing the membrane fluidity, omega-3 PUFAs can improve the binding of neurotransmitter and their receptors and ion channel function in the membrane (22, 23). It has been reported that DHA can facilitate gamma-aminobutyric acid (GABA) systems binding and increase the rate of its receptor desensitization by modulating the elasticity of the lipid bilayer (24–26).

### Stimulating cell membrane expansion and promoting membrane fusion

It has been found that by activating the plasma membrane protein syntaxin 3, omega-3 PUFAs can stimulate cell membrane expansion at the nerve growth cones, thereby promoting neurite outgrowth (27). As an enriched component in brain membrane phospholipids, DHA plays important roles in neurite outgrowth and neurotransmitter releases, the latter is a process involving membrane fusion and soluble N-ethylmaleimide-sensitive fusion factor attachment protein receptors (SNARE) complex binding or disassembly (23, 28).

### Regulatory role on signal transduction

Omega-3 PUFAs can act as agonist ligands for G protein-coupled receptor 40 (GPR40) and G protein-coupled receptor 120 (GPR120), peroxisome proliferator-activated receptors (PPARs) and retinoid X receptor α (RXRA). In addition, omega-3 PUFAs can also downregulate nuclear factor kappa-B (NF-κB) through their inhibitory effects on toll-like receptor 4 (TLR4) or binding to peroxisome proliferator-activated receptor-γ (PPARγ) (29–31). Omega-3 PUFAs may exert neurological benefits through activating corresponding receptors such as GPR120, GPR40 as well as PPARs (23). Using RXRA conditional knockout mice, unesterfied DHA has been found to promote spinogenesis, synapse formation and transmission *in vivo* in a RXRA-dependent manner (32).

### Regulating gene expression and epigenetic modifications

As previously reported, numerous brain genes expression was found to be regulated by omega-3 PUFAs supplementation (33). In addition, omega-3 PUFAs supplementation can modulate DNA methylation and histone modifications (34, 35). For example, high-mobility group box 1 (HMGB1), a nuclear regulator of gene expression, acts as an endogenous danger signal to activate inflammatory responses (36). Omega-3 PUFAs can prevent traumatic brain injury-induced inflammatory response through deacetylation of the HMGB1/NF-κB pathway.

### Antagonizing inflammation and modulating immune response

Omega-3 PUFAs and their derivatives are multifunctional regulators of inflammation (37). Omega-3 PUFAs can competitively inhibit AA metabolic enzymes by competing with AA *in vivo*, thereby inhibiting AA mediated production of inflammatory substances. Omega-3 PUFAs plays important roles as the precursor of specialized pro-resolving mediators (SPMs), such as maresins, protectins, resolvins, and lipoxins, all of which are involved in the process of inflammation resolution (38). Omega-3 PUFAs can regulate nuclear transcription factors in the nucleus to inhibit the expression of inflammatory factors. By binding to the specific receptor such as GPR120, omega-3 PUFAs can downregulate the proinflammatory signal pathways, such as NF-κB and c-Jun N-terminal kinases (JNK)-related pathways (39, 40). Omega-3 PUFAs can also inhibit TLR4 and tumor necrosis factor receptor (TNFR), thereby inhibiting the expression of pro-inflammatory factors (32). As to cellular immune response, omega-3 PUFAs can also increase the expression of the macrophage or microglia M2 phenotype, thereby promoting the resolution of inflammation (41, 42).

### Affecting mitochondrial function and reactive oxygen species homeostasis

Through changing mitochondrial membrane phospholipid composition and membrane viscosity, omega-3 PUFAs have various effects on mitochondrial function (i.e., membrane potential, respiration, individual complex activities, and ROS production). Supplementation with omega-3 PUFAs increases EPA or DHA while decreases omega-6 PUFAs in mitochondrial membrane, and can increase cardiolipin, a critical phospholipid for optimal mitochondrial function. Some literature reports that omega-3 PUFAs increases the antioxidant potential through increasing the activity of glutathione-related antioxidant enzyme and superoxide dismutase activity (43). As to the effect of omega-3 PUFAs supplementation on ROS production within the mitochondria, studies have shown inconsistent results possibly due to different experimental conditions (43–45).

### Affecting cell proliferation, cell viability, cell repair or apoptosis

Many studies have reported that dietary omega-3 PUFAs improves neural viability, promotes the proliferation of neurocytes, benefits brain cell survival and repair, and inhibits apoptosis through neurotrophic, anti-apoptotic, and anti-inflammatory signaling (46–48). In addition, DHA can increase neurogenesis through influencing cell-fate decision of adult neural stem cells and survival of the newly born cells (49).

## Effects of omega-3 PUFAs on depression

A number of epidemiological studies have shown that appropriate omega-3 PUFAs intake or higher serum omega-3 PUFAs are associated with lower risk of depression (50–52). Similarly, the depressed people exhibited lower levels of omega-3 PUFAs in blood samples than the health controls (53, 54). Furthermore, many human studies have also pointed out that the intake ratio of omega-3 PUFAs: omega-6 PUFAs is inversely associated with the risk of depressive symptoms (55, 56). Selective dietary deprivation of omega-3 PUFAs over several generations or post-weaning has consistently been shown to increase the expression of depression/anxiety-like behavior without affecting general locomotor activity in rodents. Some researchers have further suggested that omega-3 PUFAs index (which refers to the sum of EPA and DHA in red blood cells) may be used as potential treatment response marker for youthful depressed patients receiving omega-3 PUFAs (57). The expert consensus panel has reached up to consensus on using omega-3 PUFAs in the prevention and treatment of MDD subgroups such as pregnant women, children, and older adults (5).

Low levels of omega-3 PUFAs, particularly EPA, are found to be associated with depressive/anxious mood, low cognitive function, sleep disturbance, aggression and impulsive behaviors (58–61). Omega-3 PUFAs supplementation can improve many aspects of depressed patients including emotion regulation skills, cognitive function, sleep, and so on (58, 62–64). In addition, it has been reported that lower omega-3 PUFAs intake or serum omega-3 PUFAs levels are associated with greater risk of suicide attempt and MDD (65).

Several cross-sectional studies fail to find significant associations between omega-3 PUFAs and depressive symptoms (66, 67). This may mean that not all subtypes of depression are responsive to omega-3 PUFAs treatment. MDD is well recognized as a multifactorial disease which can be caused by biological, psychological or social factors. A simple lack of omega-3 PUFAs does not necessarily trigger depression, or sufficient omega-3 PUFAs can certainly avoid depression. The effects of omega-3 PUFAs on depression can be confounded by the etiology, physical constitution of patients, characteristic of fish oil (including purity, ratio of EPA to DHA), dose and duration of fish oil intake and so on.

## Possible anti-depressant mechanisms of action of omega-3 PUFAs

### Anti-neuroinflammatory effects of omega-3 PUFAs

Multiple lines of evidence have shown that there is a strong correlation between inflammation and depression. This evidence includes: (1) Depression is often accompanied by increased neuroinflammation (68). In the CNS, increased pro-inflammatory cytokines, which may come from the periphery or be produced from cells within the CNS, and activation of microglia as the resident immune cells of the brain are observed in MDD by imaging and post-mortem studies (69–73). (2) The central administration of exogenous inflammatory irritants can induce depressive symptoms (74, 75). (3) Chronic psychological or physiological stress results in neuroinflammation, which plays an important role in the occurrence of depression (76–79). (4) Neuroinflammation has close relationships with deficiency of monoamine neurotransmitter, dysfunction of brain neurotransmitters, hyperactivation of HPA axis, oxidative stress, neurodegeneration, cognitive dissonance and so on (80).

Omega-3 PUFAs and their derivates are effective in reducing neuroinflammation and has the therapeutic potential in treating neuroinflammation-related brain or mental diseases, such as Alzheimer's disease and substance abuse (81–83). In addition, it has been found that during development, deficiency of omega-3 PUFAs in diet dysregulates offspring's microglial homeostasis and increases microglial-driven inflammatory response, resulting in excessive synaptic pruning and subsequent behavioral abnormalities in mice (84).

It has been suggested that inflammation can be used as a predictive biomarker for response to omega-3 PUFAs in MDD (85). Both chronic inflammation and omega-3 PUFAs deficiency have often been found to be associated with MDD (86, 87). A number of studies have pointed out that omega-3 PUFAs (particular EPA) can probably exert some of their clinical effects *via* anti-inflammatory mechanisms of action, and patients with high inflammation levels have better improvement in depressive symptoms in response to omega-3 PUFAs supplementation (17, 88). Indirect evidences have pointed out that anti-neuroinflammatory mechanisms may be implicated in the antidepressant effects of omega-3 PUFAs (89). *In vitro* cell culture studies have shown that omega-3 PUFAs prevents the inflammatory response of microglia, which may be implicated in its antidepressant effects (90, 91). Moreover, supplementation of omega-3 PUFAs significantly reduced doxycycline (DOX)-induced neuroinflammation and effectively protected DOX-induced depressive behaviors (92).

### Role of omega-3 PUFAs in the modulation of functions of HPA axis

Hyperactivity of HPA axis, with resulting high cortisol levels is commonly found in depressed patients. Disfunction of glucocorticoid receptors (GRs) which impair the HPA axis negative feedback is one of the main causes of HPA axis hyperactivity. The brain GR, especially expressed in the hypothalamic paraventricular nucleus, hippocampus and prefrontal cortex, are generally assumed to subserve the bulk of glucocorticoid feedback regulation of the HPA axis (93).

Preclinical and clinical data has reported that low plasma omega-3 PUFAs levels have correlation with higher corticotrophin-releasing factor (CRF) (94) and higher plasma cortisol (95–97), while supplementation with omega-3 PUFAs can reduce CRF expression and corticosterone secretion (98, 99). Healthy men treated with 3 weeks of fish oil intake show a decreased cortisol response to acute mental stress (100). As to high chronically stressed men subjects, omega-3 PUFAs phosphatidylserine supplementation is also found to improve the function of HPA axis (101). In a rat model of depression, corticosterone hypersecretion induced by chronic restraint stress is dampened by omega-3 PUFAs supplementation (102). On the aspects of the CNS, evidence shows that nutritional omega-3 deficiency dampens the GR signaling pathway in the PFC of mice, which is associated with dendritic arborization in PFC as well as emotional deficits (103). It has also been shown that omega-3 PUFA supplement ameliorates the decreased expression of GR in the hippocampus of parous rats induced by the omega-3 deficient diet, which may promote the hyperactivity of the HPA axis and postpartum depression (104).

The possible mechanism of omega-3 PUFAs in regulating HPA activity may be related to the fact that omega-3 PUFAs can significantly down-regulate the expression of inflammatory factor while increasing the negative feedback sensitivity of HPA axis. Indeed, inflammatory cytokines and their related signaling pathways have been well-known to inhibit GR function, further leading to attenuated negative-feedback inhibition of the HPA axis (105, 106). The anti-inflammatory properties of omega-3 PUFAs can thereby help to reduce the irritation of inflammatory stress on the secretion of CRF and thus inhibit HPA hyperactivity (107, 108). In rat cortical cultures, DHA treatment inhibits corticosterone-induced downregulation of GR expression on βIII- tubulin-positive neurons, which may contribute to the beneficial effect of DHA on ameliorating stress-induced neuronal damage (109). Up-regulation of GR expression by omega-3 PUFAs may also be related to down-regulation the expression of miRNA218 which is a post-transcriptional regulator of GR gene expression. Studies in female rats have reported that omega-3 PUFAs supplementation improve the maternal-pup separation-induced postpartum depression and post-menopausal depression, possibly involving the effects on HPA axis activities associated with reduced miRNA-218 expression and increased GR expression in the hippocampus (107, 110).

### Anti-central oxidative stress effects of omega-3 PUFAs

Depression is often accompanied by excessive oxidative stress in the brain, which may lead to neurotoxicity and neuronal degenerative processes including decrease in neuroplasticity, neurogenesis, and an increase in apoptosis (111, 112). Many reasons including abundant O2.-/H2O2 by-products of mitochondrial respiration resulting from the brains extraordinary ATP demand, action potential dependent Ca2+ signaling-induced oxidative stress, glutamate (Glu)-induced excitotoxicity, extremely high content of unsaturated fatty acids and modest endogenous antioxidant defenses, make the brain especially vulnerable to oxidative stress (113–115). A number of the preclinical and clinical studies have reported increased oxidative biomarkers but lowered levels of antioxidants in the neurobiology of depression (116–118).

Evidence suggests that omega-3 PUFAs supplementation could attenuate oxidative stress in the brain (119–122), which might provide beneficial effects in depression prevention and treatment (51, 123). Postnatal omega-3 PUFAs supplementation can significantly enhance glutathione levels and reduce lipid peroxidation in the dentate gyrus and the cerebellum of prenatal ethanol exposure animals (124). A systematic review and meta-analysis of clinical trials have indicated that omega-3 PUFAs supplementation can enhance antioxidant defense through increasing serum total antioxidant capacity, glutathione peroxidase (GPx) activity, while reducing malondialdehyde levels (125). Oral administration of omega-3 prevents protein carbonylation and lipid peroxidation, and decreases the activity of myeloperoxidase, while improves the activities of superoxide dismutase and catalase in the brain of rats subjected to stress events (126). Using proton magnetic resonance spectroscopy, researchers found that the supplementation of 12-wk omega-3 decreases *in vivo* thalamus glutathione concentration in patients “at risk” for major depression (127). Several mechanisms can be suggested for the effect of omega-3 PUFAs on oxidative stress, including: (1) Increasing superoxide dismutase activity, elevating resistance to ROS damages and decreasing lipid peroxidation; (2) Inhibiting cyclooxygenase-2 (COX-2) enzyme activity. COX-2 metabolizes AA to inflammatory and oxidant prostaglandins which may promote lipid peroxidation; (3) Increasing the expression of nuclear factor-erythroid 2-related factor 2 (Nrf2) which is a transcriptional regulator that can effectively mediate antioxidant response by stimulating expression of the various antioxidant and anti-inflammatory genes (125, 128). In rat primary astrocytes, omega-3 PUFAs treatment can reduce ROS generation and enhance the antioxidant defense through Nrf2 activation under basal and oxidative stress conditions, suggesting that enrichment of astrocytes with omega-3 PUFAs may help to protect neurons in harmful conditions (129). Transcriptomic analyses of human hippocampal progenitor cell show that both EPA and DHA treatment regulates immune response pathways and Nrf2-mediated antioxidant pathways, which may be the molecular mechanisms underlying the preventive effect of omega-3 PUFAs on cortisol-induced decrease in neurogenesis and increase in apoptosis (130).

It has been found that only among participants with increased oxidative stress biomarkers, the omega-3 PUFAs index is negatively correlated with depressive symptoms, suggesting that oxidative stress status may be taken as a potential predictor of response to omega-3 PUFAs treatment of depression (131). Moreover, evidence indicates that omega-3 PUFAs may be more effective in improving the depressive symptoms of coronary heart disease patients with higher levels of oxidative stress marker (132). Omega-3 PUFAs can also prevent the brain's oxidative damage by decreasing the levels of protein carbonylation, lipid peroxidation, and the concentrations of nitrite/nitrate, and reducing myeloperoxidase activity, while increasing superoxide dismutase and catalase activities, which may contribute to the inhibitory effects of omega-3 PUFAs on the depressive-like behavior of the rats subjected to early or late life stress (126).

### Anti-neurodegenerative effects of omega-3 PUFAs

Neurodegenerative disorders have been closely related to depression (133). The physiological factors underlying various neurodegenerative changes include: increased inflammation level, enhanced oxidative stress damage, decreased secretion of the brain-derived neurotrophic factor (BDNF) and excessive glucocorticoid level associated with the chronic stress. The evidence of the correlation between neurodegeneration and depression can be mainly derived from the following aspects: (1) Hippocampal and the pre-frontal cortex (PFC) volume has been observed to be consistently reduced in the depressed patients (134–136); (2) Decreased levels of BDNF, dendritic atrophy, decreased neurogenesis, and increased neuronal death in patients with depression (137, 138); (3) Depression and other neurodegenerative diseases can exhibit a high co-morbidity (139). Previous epidemiological studies have shown that the co-morbidity rate of depression and Alzheimer's disease was about 40% (140), and the co-morbidity rate of Parkinson's disease was about 30% (141).

Omega-3 PUFAs play important roles in preventing neurodegeneration by inhibiting neuroinflammation, promoting synaptic plasticity and neurogenesis, facilitating nervous system repair, and protecting against the reduction of gray matter volume and the decline of white matter integrity (19, 142, 143). Studies have found that omega-3 PUFAs can promote the proliferation and migration of nerve cells, and inhibit apoptosis (48, 144). According to a ten-year follow-up study, higher levels of plasma EPA and DHA are associated with a slower decline in medial temporal lobe volume and a lower risk of dementia in older adults (145). Higher blood EPA level is found to have correlations with lower gray matter atrophy in the right amygdala, while higher atrophy of the right amygdala is correlated with more severe depressive symptoms (146).

Omega-3 PUFAs can prevent neurodegeneration through modulating a variety of pathways, including anti-apoptotic (147, 148), anti-oxidative (149–151), and anti-inflammatory pathways (152). Omega-3 PUFAs supplementation have been also found to increase the synthesis of neutrophic factor BDNF (153–155). BDNF plays important role in protecting against neurodegeneration and promoting neuronal plasticity, thereby having potential in depression treatment (156, 157). In addition, changes in the activities of telomerase and mammalian target of rapamycin (mTOR) may also be involved in the anti-neurodegeneration actions of omega-3 PUFAs (158, 159). Clinical trials have also confirmed that omega-3 PUFAs can effectively improve neurodegenerative diseases (such as Alzheimer's and Parkinson's diseases) with an associated improvement of the co-morbid depressive symptoms (160).

### Pro-neuroplastic and pro-synaptic plasticity effects of omega-3 PUFAs

Depression is deeply connected with irregular neural plasticity processes which are often found in the prefrontal cortex, hippocampus, amygdala and other limbic systems (161). Impaired neural plasticity can be primarily reflected in decreased neurogenesis, reduced dendritic spine density, decreased synapse number and strength, reduced synaptic remodeling, dendritic atrophy, as well as reward circuit dysregulation (162–166). At present, it has been found that the vast majority of antidepressant treatments, including physical therapy (such as electroconvulsive therapy, transcranial direct current stimulation, and transcranial alternating current stimulation) and drug therapy (such as fluoxetine, ketamine, and TJZL184), can primarily exert their antidepressant effects by regulating neural plasticity (167–170).

Omega-3 PUFAs have been shown to promote neuroplasticity in multiple ways. DHA supplementation could effectively promote neurogenesis by increasing the proliferation of neural stem/progenitor cells (NSPCs) as well as the number of NSPCs differentiating into the neurons and promoting the survival of newly born neurons (49). Moreover, increasing the brain levels of omega-3 PUFAs can increase the synthesis of new dendritic spines and synapses (171). The underlying mechanisms for the pro-neurogenesis effect of omega-3 PUFAs may be related to activation of proliferation-related pathways involving signaling molecular including GPR40, p38 MAPK, cAMP-response element binding protein (CREB), and BDNF (172–174). By using transgenic fat-1 mice rich in endogenous omega-3 PUFAs, researchers have found that substantial increase in brain DHA can significantly promote hippocampal neurogenesis and increase the genesis of dendritic spines of CA1 pyramidal neurons (172).

Omega-3 PUFAs supplementation could also regulate synaptic formation, synaptic transmission and affect synaptic plasticity (32, 171, 175). Omega-3 PUFAs may play an important role in regulating the expression of several important neural and glial proteins such as E-cadherin, early growth response 1, postsynaptic density protein 95, and signaling factors that have been implicated in synaptic plasticity [such as N-methyl-D-aspartic acid (NMDA) receptor and Fyn] (176). It has been reported that omega-3 PUFAs deficiency can reduce long-term potentiation (LTP), the concentrations of glutamate receptor subunits, and synaptic vesicle proteins at the hippocampal glutamatergic synapses (177).

In addition, DHA and EPA can be converted into endocannabinoids (eCBs) docosahexaenoyl ethanolamide (DHEA) and eicosapentaenoyl ethanolamide (EPEA) which exerts physiological effects through activating eCB receptors. DHEA and EPEA have been reported to exhibit various immunomodulatory and anti-inflammatory activities and effects on food intake and mood (20, 178). The eCBs can induce short-term changes and long-term synaptic plasticity in the whole nervous system, because eCBs synthesized in the postsynaptic neurons can function reversely to regulate the presynaptic input (179). Maternal omega-3 PUFAs deficiency can induce the impairment of eCBs gating of LTP in hippocampus of weaned pups (180). It has been also found that life-long omega-3 PUFAs deficiency can lead to the specific inhibition of eCBs-mediated long-term synaptic depression in the prelimbic prefrontal cortex and the accumbens of adult mice. This effect is accompanied by decreased CB1 receptor function which is associated with impaired emotional behavior (181).

### Neurotransmitter system modulatory effects of omega-3 PUFAs

The neurotransmitter and neurotransmitter receptor hypothesis of depression propose that various disorders in multiple neurotransmitter systems are involved in etiopathology of depression. Emerging literature has also shown that depression might be associated with the various molecular abnormalities and functional deficiency in brain transmitter systems including that in 5-hydroxytryptamine (5-HT), dopamine (DA), norepinephrine (NE), Glu and GABA (157). Accumulating evidences indicate that the neurotransmitter transmission predominantly depends on adequate level of omega-3 PUFAs or optimal omega-6 PUFAs: omega-3 PUFA ratio in the brain (23). Being rich in membranes of the synaptic terminals, DHA has been considered to be important for the function of neurochemical transmission. Low availability of omega-3 PUFAs can influence the synthesis, synaptic release, uptake of multiple neurotransmitters including 5-HT, DA, NE, Glu and GABA.

Accumulating evidences indicate that the normal functional activities of neurotransmitter systems depend on adequate levels of omega-3 PUFAs in the brain (23, 182). Omega-3 PUFAs have wide effects on the synthesis, synaptic release, uptake of multiple neurotransmitters including 5-HT, DA, NE, Glu and GABA (23, 183, 184). It has been pointed out that improving the transmission of 5-HT and DA; reducing 5-HT2 receptor and increasing D2 receptor in the frontal cortex may be the possible mechanisms underlying the beneficial effects of omega-3 PUFAs on depression (23). It is reported that fish-oil supplementation produces an antidepressant-like effect in LPS-induced depression model and this effect is related to decreased expression of indoleamine-2,3-Dioxygenase and elevated 5-HT levels in the hippocampus (185). Omega-3 PUFAs can also influence the expression levels of multiple neurotransmitter receptors. It has been shown that DHA supplementation can prevent the increase of binding density of 5-TH receptors (5-HT1A and 5-HT2A), CB1 and GABA-A receptors induced by the high saturated fat diet, which have been related to the cognitive function of the brain (186).

In addition, previous studies have demonstrated that omega-3 PUFAs deficiency can aggravate the age-associated decrease in glutamatergic synaptic efficacy in the hippocampal CA1 (187). It can further affect the glutamatergic synapse development and anxiety-like behavior in male adult rats (188). It has also been found to decrease the subunits of NMDA receptors NR2A, NR2B in rodents (188–190). Similarly, omega-3 PUFAs deficiency also leads to decreased concentrations of Glu receptor subunits (GluA1, GluA2 and NR2B) and other synaptic vesicle proteins in the hippocampal synaptosomes of mice (177).

Preclinical and clinical findings have suggested that eCBs/CB1R signaling can contribute to depression risk and omega-3 PUFAs can exert the anti-depressant effects through altering the PUFA-derived eCBs levels in the whole brain (181, 191–193). It has been reported that omega-3 PUFAs supplementation can increase plasma DHEA and EPEA levels and increased EPEA levels are positively related to the clinical remission rate of MDD patients (180).

### The correlations of the six mechanisms of omega-3 PUFAs action

These six aspects of omega-3 PUFAs action may not play independent parts, but just like different aspects of the same thing they probably work in an interconnected system. The simplified model for the assumed interconnection of the six mechanisms are shown in Figure 1. Neuroinflammation and oxidative stress often flame each other and have been considered as the major causes of neurodegeneration followed by MDD (194). Chronic stress-induced hyperactivation of the HPA-axis and neuroinflammation can create a vicious cycle, lead to dysfunction of neurotransmitters system, impair neuroplasticity and promote neurodegeneration (195–199). It has been indicated that HPA axis hyperactivation causes inflammation response in MDD. Immunological communication between the CNS and the body periphery triggers neuroinflammation, which further induces the failure of glucocorticoid negative feedback within the brain. In addition, inflammatory factors activate the kynurenine pathway resulting in the reduction of serotonin biosynthesis and the increased production of neurotoxic metabolites, and eventually neurodegeneration (198). There are lots of papers which have reviewed the correlations between neuroinflammation and neural or synaptic plasticity, neuroinflammation and neurotransmitter systems, neurotransmitter systems and synaptic plasticity, which can be referred to the following references (200–203). We also summarized the experimental designs and results of animal studies reported in the references in this paper. As shown in Table 1.

**Table 1:** Experimental design and results summary of animal studies in references.

**References**	**Year**	**Animals**	**Experimental model**	**Omega-3 Dose**	**Effects**	**Molecular change**
Song et al. (95)	2009	Sprague Dawley rats	Olfactory bulb resection depression model	1% EPA diet	Water maze: spatial memory↑	mRNA expression and activity of cPLA2↓; Serum IL-1β and PGE2 concentrations↓; CRF mRNA expression and blood corticosterone concentration↓; NGF expression in the hippocampus↑;
Ferraz et al. (102)	2011	Wistar rats	Restraint stress induced depression model	3.0 g/kg animal weight of an oral compound containing 12% of EPA and 18% of DHA	FST: immobility frequency↓; swimming frequency↑; climbing frequency↑; Water maze test: mean latency time↓; percentage of spent time in target quadrant↑; percentage of entries into closed arms↓; EPM: percentage of entries into open arms↑; percentage of time spent in closed arms↓; percentage of time spent in the open arms↑	None
Labrousse et al. (204)	2012	C57Bl6/J mice	Control diet non-depression model	An isocaloric LCω3 PUFA supplemented diet containing a mixture of rapeseed oil, high-oleic sunflower oil, palm oil and tuna oil resulting in a 10% EPA and 7% DHA diet	Spatial recognition: Spatial memory deficit↓	AA/dGLA↑; EPA and DHA in the brain↑; (dGLA+EPA)/AA↑; microglia-dependent activation↓; proinflammatory cytokines production in microglia↓; CD11b mRNA expression↓; TNF-α expression mRNA↓; IL-6 mRNA expression↓; IL-1β expression↓; length of astrocytic processes in aged mice↑; c-Fos positive cells↑; microglia-dependent activation↓; proinflammatory cytokines production↓
Balvers et al. (193)	2012	C57BL/6 mice	i.p. LPS induced depression model	1% or 3% fish oil	None	DHEA↑; EPEA↑; endocannabinoids↓; NAEs↓; DGLEA↓; adipose tissue levels of SEA↑; plasma levels of SEA↓; ARA↓; DHA↑; EPA↑; oxylipins↓; LTB4 in ileum and adipose tissue↓; LTB4 in liver↑; Lipoxin A4↑
Larrieu et al. (205)	2014	C57BL/6 mice	Chronic social defeat stress induced depression model	3.1% lipids	Number of social explorations↓ OFT: time spent exploring the center↓	Simplification of apical dendritic tree on pyramidal neurons of the dlPFC and dmPFC↓; total corticosterone elevation↓; HPA axis hyperactivity↓; neuronal atrophy↓
Wu et al. (92)	2016	Sprague Dawley rats	Depressive model induced by intraperitoneal injection of doxorubicin	EPA: DHA 3:2; EPA 510mg/kg; DHA 360mg/kg	Weight loss↓ OFT:Number of crossings↑; number of rearing↑; latency time↓; FST: swimming time↑; immobility time↓	MDA in prefrontal cortex↓; MDA in hippocampus↓; SOD in hippocampus↑; IL-1 mRNA expression in prefrontal cortex↓; IL-6 mRNA expression in prefrontal cortex↓; IL-6 mRNA expression in hippocampus↓; TNF-α mRNA expression in hippocampus↓; protein level of NF-κB↓; protein level of iNOS↓; Number of nuclear pyknosis↓; Apoptotic index TUNEL-positive cells↓gene expression of Bcl-xl↑; gene expression of Bcl-2↓.
Larrieu et al. (103)	2016	C57BL6/J mice	Mifepristone subcutaneous implantation non-depression model	Containing 6% of rapeseed oil	Number of social interaction↑; OFT: Center time ↑	Plasma corticosterone levels ↓; the total apical dendritic material in both dlPFC and dmPFC ↑
Abdel-Maksoud et al. (153)	2016	Sprague-Dawley rats	Control diet non-depression model	EPA: DHA 3:2; EPA 180mg; DHA120mg	None	BDNF gene expression↑; serum total cholesterol↓; triacylglycerol↓; serum glucose level↓; HOMA index↓; triacylglycerol levels↓
Morgese et al. (98)	2017	Wistar rats	Control diet non-depression model	Containing 6% total fat in the form of only rapeseed oil (n-3 enriched, rich in linolenic acid 18:3n-3)	FST: immobility frequency↓; swimming frequency↑; struggling frequency↑; OFT: time of performing self-grooming↓	Cortical 5-HT concentrations↓; CRF content↑; corticosterone levels↑; plasmatic Aβ levels ↑; NA↑
Tang et al. (104)	2018	Sprague-Dawley rats	Control diet non-depression model	Fish oil (20 g/ kg)	FST: immobility frequency↓; SPT: sucrose preference↑	Protein expressions of glucocorticoid receptor ↑
Cigliano et al. (206)	2019	MRL/lpr mice	Non-depression model	An oral dose (30 mg) of FO (85%) containing 16 and 9,5 mg of DHA and EPA	None	Double-stranded DNA (anti-dsDNA) IgGs↓; TNF-α↓; PPAR-γ↑; DHA concentration in the brain↑; BDNF↑; SynaptophysinI↑; SynaptotagminI↑; SynapsinI↑; compensatory hyperactivation of phase 2 enzymes (GSR, G6PD) activities↓; GCL↓; GSR mRNA levels↓; Nrf2 ↓
Yang et al. (183)	2019	Sprague-Dawley rats	CUMS induced depression model	Fish oil (20 g/kg)	FST: Immobility times↓; SPT: sucrose preference↑; OFT: number of locomotor crossing↑; number of rearing↑	5-HIAA↓; DOPAC↑; HVA↓; VMA↓; GLN↑; DA turnover rate 2↓; NE turnover rate 1↓; NE turnover rate 2↓; DA/NE between-metabolite ratio 1↑
Choi et al. (107)	2020	Wistar rats	Pup separation-induced depression model	EPA:DHA 5:3; EPA 450mg; DHA260mg	FST: immobility time↓; climbing time↑; Sucrose preference index↑; Pup retrieval test: Latency of the first contact↓; Latency to retrieve↓	Adrenocorticotropic hormone↓; corticosterone↓; hypothalamic corticotrophin releasing factor↓; hippocampal miRNA-218↓; prostaglandin E2↓; TNF-α↓; IL-6↓; miRNA-155↑; serotonin↑; serotonin-1A receptor↑; cAMP response element binding protein (CREB)↑; pCREB; brain-derived neurotrophic factor↑; miRNA-182↑.
Cutuli et al. (144)	2020	C57BL/6 mice	icv. mu-p75-saporin induced depression model	EPA: DHA 5:4 300 mg/kg	EPM: expected aversion↓; NORT: total object contact time↓	Preserved hippocampal volume; neurogenesis in the dentate gyrus↑;astrogliosis in the hippocampus↓
Peng et al. (207)	2020	Sprague Dawley rats	CUMS induced depression model	1% ethyl-EPA (96% pure) or 1% DHA (96% pure)	SPT: Sucrose consumption↑; FST: immobility time↓; OFT: numbers of locomotor crossing↑; numbers of rearing↑	Arachidonic acid (AA) level in the brain↓; docosapentaenoic acid in the brain↑; total cholesterol level ↓; serum corticosterone ↓; NE ↑; 5-HT ↑; NE/MHPG↑; IL-1β↓; IL-6↓; TNF-α↓; CD11b expression↓; p75NTR expression↓; GDNF expression↑; NF-KB and p38 expression↓; bax expression↓; bcl-2↑; bax/bcl-2↓
Carabelli et al. (185)	2020	Wistar rats	i.p. LPS induced depression model	3.0 g/kg (approximately 3.0 mL/kg) of fish oil containing 18% of EPA and 12% of DHA	Weight loss↓; FST: swimming frequency↑; immobility time↓	5-HT↑; 5HIAA/5-HT↓; IDO expression↓
Choi et al. (110)	2021	Wistar rats	CMS+ovariectomy induced depression model	EPA: DHA 3:2; EPA 300mg/kg; DHA260mg/kg	FST: swimming time↑; immobility time↓; SPT: sucrose preference index↑	Brain endocannabinoid/oxylipin levels↑; blood levels of adrenocorticotropic hormone and corticosterone↓; tumor necrosis factor-α, interleukin (IL)-6, IL-1β, and prostaglandin E2↓; brainstem serotonin levels and hippocampal expression of the serotonin-1A receptor, cAMP response element-binding protein (CREB), phospho-CREB, and brain-derived neurotrophic factor↑

“↑” Indicates increased levels or protein expression of factor substances *in vivo* after treatment with omega-3 compared to controls. “↓” Indicates decreased levels or protein expression of factor substances *in vivo* after treatment with omega-3 compared to controls. FST, Forced Swimming Test; SPT, Sucrose Preference Test; OFT, Open Field Test; EPM, Elevated Plus Maze; NORT, New Object Recognize Test; NGF, Nerve growth factor; LTB4, Leukotriene B4; MDA, Malondialdehyde; HOMA index, Homeostatic model assessment index; HVA, Homovanillic acid; VMA, Vanillylmandelic acid; GLN, glutamine.

According to research results, omega-3 PUFAs deficiency often leads to dysfunction of multiple neurobiological systems, such as neuroinflammation, inactivated GR signaling pathway and HPA axis hyperactivity, deteriorated serotoninergic, noradrenalinergic and dopaminergic neurotransmission, impaired neurogenesis, neurodegeneration and so on (96, 103, 208). It has been found that chronic dietary omega-3 PUFAs deficiency led to a significant reduction in 5-HT and NA content, increased production of kynurenine, along with HPA axis hyperactivity, higher proinflammatory cytokine production associated with higher expression of TLR2 and TLR4 and increased expression of oligomeric Aβ in hippocampus of female rats (209).

Both clinical and pre-clinical evidences have shown that the alterations of many different aspects of the CNS are involved in the anti-depressant effect of omega-3 PUFAs supplementation (63, 88, 89, 130, 184, 205, 206, 208, 210–213). Therefore, the six antidepressant mechanisms of omege-3 PUFAs that we summarized probably act synergistically but not separately. However, there is currently no clear conclusion on how these mechanisms are linked or how they interact, and whether there are causal links. All these questions require further studies to be answered.

Based on existing evidence, we summarized a variety of central mechanisms of omega-3 PUFAs anti-depressant actions, from molecular mechanisms to cellular mechanisms to neurobiological mechanisms. **At the molecular level**, omega-3 PUFAs directly changes lipid profiles, regulating membrane fluidity and membrane-associated cellular processes such as the assembly of membrane protein complexes and lipid valves; neurotransmitters and neuroendocrine exocytosis as well as microglia phagocytosis. Through metabolism and acting on the plasma membrane or intracellular receptors, omega-3 PUFAs can improve total antioxidant capacity; prevent lipid peroxidation: produce anti-inflammatory metabolites and regulate inflammatory signal pathways. **At the cellular level**, omega-3 PUFAs indirectly affect a wide range of cellular activities, such as the cell resistance to stress damage; various signaling transduction pathways; the neurotransmitter systems; the whole-genome expression profile; cell proliferation, differentiation, growth, development, senescence, apoptosis and so on. Based on its molecular and cellular mechanisms **at the neurobiological level**, omega-3 PUFAs exert a widespread and far-reaching influence on the mood regulating function of the central nervous system. Omega-3 PUFAs can improve neuroinflammation; dysfunction of neuroendocrine (HPA axis); oxidative stress; neurodegeneration; neuroplasticity; neurotransmitters system and so on. All these effects may play roles in the prevention and improvement of depression. Abbreviations: TRPV4, Transient receptor potential vanilloid 4; ROS, Reactive oxygen species; EGFR, Epidermal growth factor receptor; GPR120,G-protein coupled receptor 120; PPARγ, Peroxisome proliferator-activated receptor-gamma; TLR4, Toll-like receptor 4; COX-2, Cyclooxygenase-2; BDNF, Brain-derived neurotrophic factor; CREB, cAMP-response element binding protein; PPAR, peroxisome proliferator-activated receptor; Nrf2, nuclear factor-erythroid 2-related factor 2; 5-HT, 5-hydroxytryptamine; NE, Norepinephrine; CB, Cannabinoids; GC, Glucocorticoid; NF-κB, nuclear factor kappa-B; JNK, c-Jun N-terminal kinases; p38MAPK, p38 mitogen-activated protein kinase.

## Future directions

The pathological mechanisms of depression have been found to be closely associated with multiple aspects of neural functions. Currently, omega-3 PUFAs are a kind of molecules of diverse biological activities, capable of producing multiple antidepressant effects in the CNS. The six possible mechanisms summarized in this review are probably interconnected in complex manner and can function synergistically to produce the anti-depressant effects. However, there is a critical need for well-designed systematic researches to identify the eventual unitary anti-depression mechanism from various different actions of omega-3 PUFAs.

Although there have been numerous studies published that have demonstrated the regulation of various physiological functions by omega-3 PUFAs in depressive disorders, there are still insufficient reports related to the causal mechanisms of omega-3 PUFAs anti-depressant action. Omega-3 PUFAs can have diverse regulatory effects on neurogenesis, synaptic plasticity, oxidative stress, neuroendocrine, and neurotransmitter transduction, but the specific molecular regulatory pathways remain largely unclear. In addition, it is also unclear to how these PUFAs affect depressive symptoms from the molecular to the behavioral level. To answer this question may depend on the thorough understanding about the role of omega-3 PUFAs in human life as well as the pathophysiological nature of depression.

It is still unclear which ones are direct or indirect mechanisms; which ones are the primary effects and which ones are the secondary mechanisms of the anti-depressant effect of omega-3 PUFAs. Clarifying these issues which will help establish the central pharmacological action and pharmacodynamics of omega-3 PUFAs.

In order to facilitate translation to application, future research may need to elucidate the anti-depressant mechanism of omega-3 PUFAs action by using quantitative systems pharmacology and to identify the clinical biomarkers and the antidepressant-response biomarkers in target subgroups of depressed patients.

Moreover, omega-3 PUFAs may play different roles in different depressed patients with different constitutions. Further studies should be conducted to explore the potential different mechanisms of the action of omega-3 PUFAs on depression in children, adolescents, postpartum women, and eldly adults or depressed patients with concomitant physical diseases such as cardiovascular disorders. This will help the personalized application of omega-3 PUFAs in different subgroups of MDD.

In addition, according to ISNPR's 2019 practice guideline for the assisted treatment of depression with omega-3 PUFAs, omega-3 PUFAs are found to be more effective as an adjuvant treatment than monotherapy for MDD treatment. So, what are the specific mechanisms by which omega-3 PUFAs can accelerate or enhance effects of other antidepressants? This requires further studies to explore the exact mechanisms underlying greater efficacy of clinical antidepressants in combination with omega-3 PUFAs. This may be the good news for clinical use of antidepressants that act quickly but are often associated with side effects, or work too slowly, or do not work significantly.

## Conclusions

This article provides a review of the neurobiological mechanisms underlying the antidepressant effects of omega-3 PUFAs. Based on the accumulated evidence from recent publications, we identified six potential mechanisms, including: (1) anti-neuroinflammatory; (2) anti-oxidative stress; (3) modulation of HPA axis; (4) anti-neurodegeneration; (5) neuroplasticity and synaptic plasticity; and (6) modulation of neurotransmitter systems. All these antidepressant mechanisms may be based on the molecular action and cellular effects of omega-3 PUFAs, however, how these processes work remains largely unknown. Although neurobiological mechanisms are probably interconnected and interdependent, the multiple sites of action of omega-3 PUFAs are still needed to be clarified. This review contributes to a better understanding the potential mechanisms of benefit of omega-3 PUFA and may provide useful references for the development of new strategies for the treatment of depressive disorder with omega-3 PUFAs.

## Author contributions

J-TZ was responsible for the study conception and design and the writing and revising of the manuscript. LZ performed the collection, the writing of the manuscript, and provided the technique support. BL made revisions and polishing to the paper and provided the funding support. J-YX and Y-QC made material support and grammar checking. All authors contributed to and have approved the final manuscript.

## Funding

This study was supported by Scientific Research Project of Education Department of Hubei Province (B2021048).

## Conflict of interest

The authors declare that the research was conducted in the absence of any commercial or financial relationships that could be construed as a potential conflict of interest.

## Publisher's note

All claims expressed in this article are solely those of the authors and do not necessarily represent those of their affiliated organizations, or those of the publisher, the editors and the reviewers. Any product that may be evaluated in this article, or claim that may be made by its manufacturer, is not guaranteed or endorsed by the publisher.
